# Life Table Evaluation of Survival and Reproduction of the Aphid, *Sitobion avenae*, Exposed to Cadmium

**DOI:** 10.1673/031.012.4401

**Published:** 2012-03-29

**Authors:** Huan-Huan Gao, Hui-Yan Zhao, Chao Du, Ming-Ming Deng, Er-Xia Du, Zu-Qing Hu, Xiang-Shun Hu

**Affiliations:** Institute of Plant Protection, College of Plant Protection, Northwest Agriculture and Forest University, State Key Laboratory of Crop Stress Biology in Arid Areas, Yangling, Shaanxi 712100, China

**Keywords:** heavy metal, wheat, fecundity

## Abstract

The effects of cadmium (Cd) on the development, fecundity, and reproduction of the grain aphid, *Sitobion avenae* Fabricius (Hemiptera: Aphididae) were estimated by constructing a life table of *S. avenae* exposed to Cd. The concentrations of Cd in the soil were as follows: 0, 10, 20, 40, 80, and 160 mg/kg. The correlation analysis of the Cd concentration in soil and wheat revealed that the amount in the wheat increased with the increase of Cd concentrations in soil. The results indicated that, the latter part of the reproduction period was significantly affected by Cd, according to the curve of the total survival rate (*l_x_*). The net reproductive rate (*R*_0_), innate capacity of increase (*r*), and finite rate of increase (*λ*) of *S. avenae* all decreased under the stress of Cd, and were lowest at a Cd concentration of 20 mg/kg. Cd also negatively affected fecundity and *m_x_* (the number of offspring produced by an individual female). At 20 mg/kg, the decline of them was most obvious. In conclusion, survival and reproduction of *S. avenae* were inhibited under the treatment of the heavy metal Cd. *Sitobion avenae* was more sensitive to Cd at concentration of 20 mg/kg compared to the other concentrations. This concentration can be used to examine the mechanisms behind population genetics and biological mutation of *S. avenae* when exposed to heavy metal.

## Introduction

The concentrations of trace metals in the topsoil can increase with the addition of chemical fertilizers, pesticides, and industrial sewage (Raven and Loeppert 1997). Heavy metal contamination from these sources has become an important global environmental problem. The transfer and accumulation of heavy metals along the food chain accelerates the deterioration of the ecological environment and influences the metabolism and development of organisms in various ecosystems (Wang et al. 2005). For example, *Tetrix tenuicornis* collected from a polluted area contained concentrations of copper, zinc, lead, and cadmium (Cd) many times higher than insects of the same species collected from unpolluted areas (Warchałowska-Sliwa et al. 2005). The transfer and accumulation of heavy metals in different organisms along the food chain also has a potential impact on the development and metabolism of phloemfeeding insects. The potential for the uptake of heavy metals by aphids was demonstrated by Crawford et al. (1995), who observed the uptake and accumulation of Cd in the black bean aphid, *Aphis fabae*, indicating a potential transfer route of Cd from wheat to aphids. Cd (Merrington et al. 1997) and zinc (Merrington et al. 1997; Green et al. 2003) can undergo bioaccumulation in the grain aphid, *Sitobion avenae*.

Developmental period, weight, fecundity, mortality, and insect population number are also negatively affected by heavy metals (Ruohomaki et al. 1996; Mousavi et al. 2003; Wu et al. 2003; Hayford and Ferrington 2005). Additionally, it has been reported that accumulation of both Pb and Cu in different host plants could result in a significant number of deviations from bilateral symmetry in the cabbage aphid *Brevicoryne brassicae* (Görür 2006). The intrinsic rate of increase (*r*), the finite rate of increase (λ), and the net reproductive rate (*R_0_*) of *S. avenae* decrease and the mean generation time (*T*) has been shown to increase under exposure to the heavy metal zinc (Zhang and Zhao 2009). Moreover, the effects of heavy metals on insects depend on the concentrations and kinds of those heavy metals. The influence of parasitism rate, time from parasitism to pupation, pupal duration, adult emergence rate, and adult female longevity of *Micrelaps bicoloratus* are intensified with the increase of zinc concentrations in diets (Xia et al. 2006). Similarly, Cd at high concentrations reduced population growth rate of the pea aphid (Kramarz and Stark 2003). However, the mean generation time of *S. avenae* is prolonged at the low concentration of zinc and shortened at high concentration. There is no significant influence on the mean generation time for *S. avenae* under the treatment of copper (Zhang and Zhao 2009).

*Sitobion avenae* is one of the most serious pests attacking agricultural plants and vectoring debilitating plant viruses (Blackman and Eastop 1984; Oerke 1994). The environment plays an important role in the course of biological evolution as a selective force that will act on the species and influence the genetic capacity of the species to respond (Harrington et al. 2001). Therefore, the influence on survival and reproduction of *S. avenae* exposed to heavy metal needs to be examined. As for *S. avenae*, the effects of zinc, copper, and lead have been reported in previous studies. However, the effects of *S. avenae* exposed to Cd have not been examined; it is not known whether or not there are similar effects on aphids among different kinds of heavy metals. Thus, this study will examine the effects of *S. avenae* exposed to varying concentrations of Cd. Results lay the foundation for research on the mechanisms of adaptation and evolution in *S. avenae* and can be utilized for the management of aphids.

## Materials and Methods

### Sitobion avenae

Individual grain aphids, *S. avenae*, were collected from a field of wheat, *Triticum aestivum* L. (Poales: Poaceae) in the district of Yangling, Shaanxi province, central China, in April 2010. One wingless adult aphid was reared on wheat plants for four to five consecutive generations at 18–20 ± 0.3 °C, 60% RH, and 14:10 L:D in a climate-controlled chamber. The population at that time was a monoclonal colony (Du et al. 2007) that was used as a source for all aphids in all laboratory experiments.

### Treatment with cadmium

Using to the concentrations of Cd (0, 200, 400 mg/kg) adopted by Kramarz and Stark (2003), with some modifications, the experimentally contaminated wheat was planted in a plastic pot (9 × 9 × 10 cm) with contaminated soil exposed to cadmium (Cd) as CdCl_2_·2.5H_2_O at five concentrations: 10, 20, 40, 80, and 160 mg/kg. The dried soil in each pot weighed 1 kg. Non-contaminated soil was used to plant the control plants. Ten wheat seeds were planted in each pot.

### Determination of Cd concentrations in wheat

At the two to three-leaf (code 12 to 13) (Zadoks et al. 1974) stage of the host plant, to determine Cd concentrations, 1 g of fresh wheat leaf tissue was homogenized with a mortar and pestle and digested with a mixture of HNO_3_/HClO_4_ (3:1 (v/v)) in each treatment for three replicates. Cd was determined by flame atomic absorption spectrophotometry (Hitachi Z-2000, www.hitachi.com) as described by Sun et al. (2005).

### Life table study

When the host plant reached stages 12 to 13, first instar nymphs were placed on the experimental plants. When the second generation of aphids began to reproduce nymphs, 30 first instar nymphs from the monoclonal colony were placed individually on 30 plants (three pots), respectively. Each individual was maintained in a small mesh bag attached to the leaf using a clip cage (0.6 cm in diameter and 0.3 cm in height). Aphids used for the experiment were reared under the laboratory conditions described above. Survivorship and daily nymphoposition of *S. avenae* adults were recorded from birth to death for each aphid in the experiment. Time-specific life table parameters of aphids were generated to calculate the total survival rate, fecundity, development duration (*T*), net reproductive rate (*R_0_*), innate capacity of increase (*r*), and finite rate of increase (*λ*).

### Experimental design and data analyses

The current study utilized the randomized complete block design. The Cd levels were considered as the treatment factors, and each of the three replications was arranged as an experimental block in a growth chamber where the experiment was conducted. The raw data from the different treatments were analyzed using TWOSEX-MSChart (Chi 2006) based on the age-stage, two-sex life table theory (Chi and Liu 1985; Chi 1988). Accordingly, the age-specific survival rate (*l_x_*) and age-specific fecundity (*m_x_*) were calculated. The fecundity was the mean of accumulated offspring. The intrinsic rate of increase (*r*) was estimated according to the iterative bisection method from the Euler-Lotka formula:
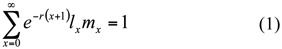


The finite rate of increase *λ* was calculated as *λ* = *e^r^*. The net reproductive rate (*R_0_*) was calculated as the mean number of offspring that an individual can produce during its lifetime. The mean generation time (*T*) was defined as the period that a population needs to increase to *R*_0_-fold of the number at the stable age-stage distribution. The formulae for *R*_0_ and *T* are the following:
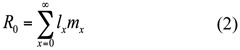

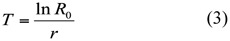


The quadratic regression analysis was carried out between the soil Cd concentration and the total survival rate of *S. avenae*, between the soil and wheat Cd concentration with quadratic regression of SPSS 17.0 software. The data for *r, λ, R*_0_ and *T* from different treatments were first examined for normality and homoscedasticity, then were tested using one-way analysis of variance (ANOVA, α = 0.05) and Student-Newman-Keuls (SNK) multiple comparisons with SPSS 17.0 software.

## Results

### Cd Concentrations in the wheat

**Figure 1.**
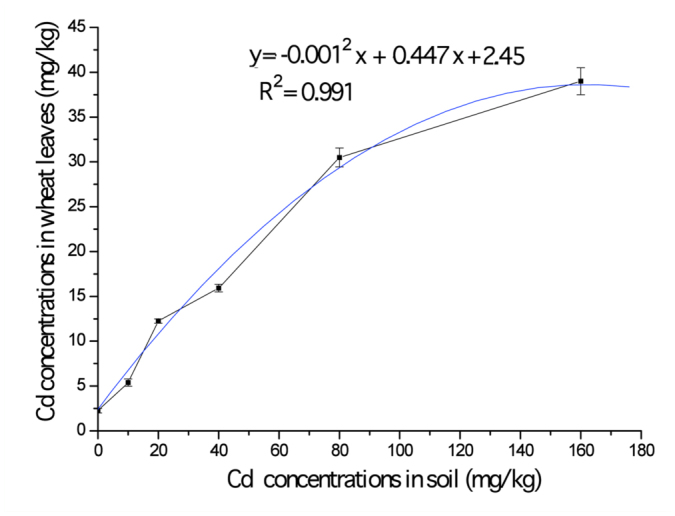
Analysis of quadratic regression between the soil and wheat leaves Cd concentration. The parameters y and x are the concentration of Cd in wheat and soil separately. The quadratic equation and coefficient are seen in the figure. High quality figures are available online.

The concentrations in the leaves of wheat consistently increased with increasing Cd levels in soil (*F*_5,17_ = 347.036, *p* < 0.05) (Figure 1). Cd level in the plant leaves, however, was reduced compared to the Cd level in the soil. The quadratic regression equation was also established through correlation analysis of the Cd concentration in soil and wheat leaves (*F*_2,3_ = 167.484, *p* < 0.01). The nonlinear relation (R^2^ = 0.991) was significant between the concentrations of Cd in soil and wheat. It could be concluded that the concentrations in the leaves of wheat nonlinearly increased with increasing Cd levels in soil.

**Figure 2.**
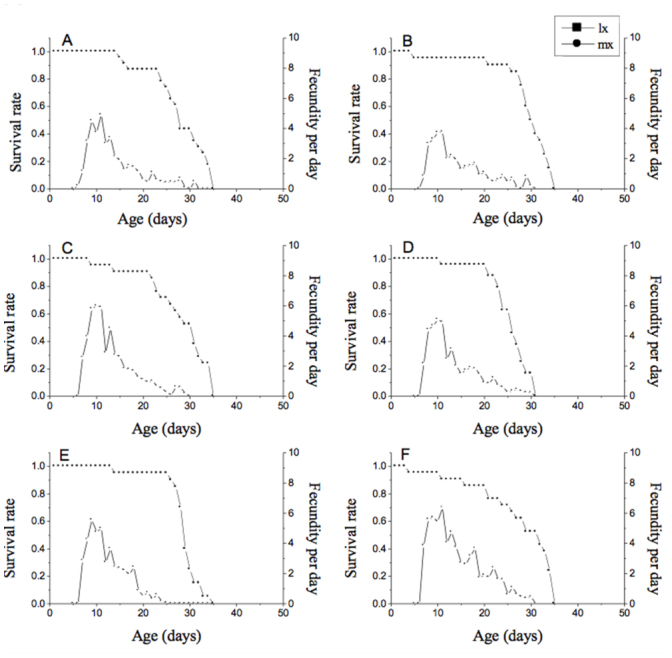
The age-specific survival rate (*l_x_*) and age-specific fecundity (*m_x_*) of *Sitobion avenae* with six levels of Cd treatment. *S. avenae* reared on the experimental contaminated wheat planted in the soil with five concentrations: 10 (A), 20 (B), 40 (C), 80 (D), and 160 mg/kg (E). The control (F) was 0 mg/kg. High quality figures are available online.

**Figure 3.**
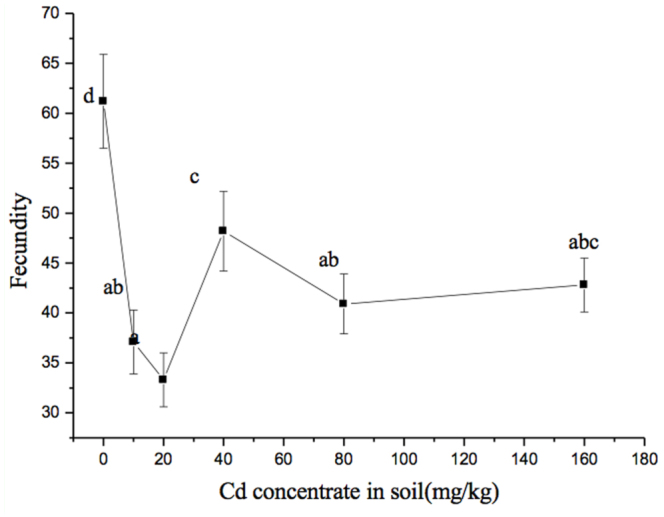
Fecundity of adult *Sitobion avenae* with six levels of Cd treatment. Different letters (a–d) indicate a significant difference (SNK test: *p* < 0.05, following one-way ANOVA). High quality figures are available online.

### Total survival rate

The total survival rate (*l_x_*) is the probability that a first instar nymph will survive to age *x*. The total survival rate of *S. avenae* declined evenly and slowly (Figures 2A–F). The rapid decrease in survival rate in the later life stages is shown in the curves under the treatment of Cd (*F*_5,17_= 175.46, *p* < 0.01) (Table 1). The parameter b in the quadratic regression equation of Cd treatments increased significantly compared to the control. This shows that exposure to Cd had significant negative effects on the later stages of reproduction.

### Fecundity

The mean fecundity of *S. avenae* with six levels of Cd treatment are shown in Figure 3. The total fecundity (mean ± SE) of *S. avenae* was 37.1 ± 3.2, 33.3 ± 2.7, 48.2 ± 4.0, 40.9 ± 3.0, and 42.8 ± 2.7 total offspring from 10 to 160 mg/kg of Cd treatments, all of which were significantly lower than the control (61.2 ± 4.7) (*F*_5,17_ = 27.150, *p* < 0.01). The value was lowest at 20 mg/kg Cd.

The age-specific fecundity (*m_x_*, the number of offspring produced by female *S. avenae* every day) is shown in Figure 2. From the control (6.37 ± 0.06), the peak values in the *m_x_* curve of aphids for Cd concentrations of 10, 20, 40, 80, or 160 mg/kg were reduced to 4.52 ± 0.01, 3.78 ± 0.52, 5.90 ± 0.49, 5.08 ± 0.21, and 5.55 ± 0.48 offspring/day, respectively (*F*_5,17_ = 469.64, *p* < 0.01) (Figure 2), which was in accordance with the change in total fecundity (Figure 3). Although the lower *m_x_* was not seen for *S. avenae* exposed to 160 mg/kg Cd compared to that of control, the total fecundity was still negatively affected by Cd because the reproduction period was reduced to 25 ± 0.34 days (Figure 2E) from the control (30.90 ± 0.49 days; Figure 2F) (*F*_5,17_ = 64.62, *p* < 0.01). *S. avenae* was more sensitive to Cd concentrations of 20 mg/kg than to the other treatments.

**Table 1.**
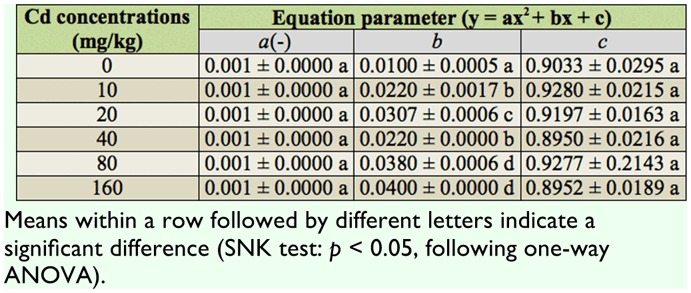
Mean ± SE of quadratic regression equation parameters of total survival rate in *Sitobion avenae* with six levels of Cd treatment.

**Table 2.**
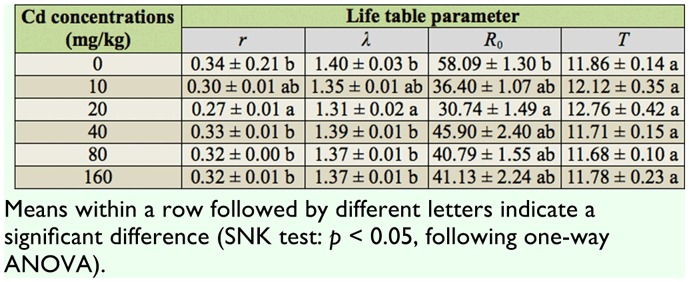
Mean ± SE of population parameters in a cohort life table of *Sitobion avenae* with six levels of Cd treatment.

### *R_0_, r*, λ, and *T*

The population parameters of the cohort life table for *S. avenae* in different Cd treatments are shown in Table 2 (*R*_0_, *F*_5,17_ = 3.612, *p* < 0.05; *r*, *F*_5,17_= 5.071, *p* < 0.01; λ, *F*_5,17_= 4.991, *p* < 0.05). Under different treatments, the values of *R_0_, r*, and λ decreased until the concentration increased to 20 mg/kg and then increased thereafter, but they were all lower than the control overall. At a Cd concentration of 20 mg/kg, the values of *R_0_, r*, and λ of *S. avenae* were significantly different from those of the control. It was concluded that Cd inhibited the rate of increase of *S. avenae* in this specific environment or would affect population increase in the long run. However, statistical analysis revealed the absence of significant differences between the values of the mean generation time (*T*) under the control and those of *S. avenae* exposed to Cd.

## Discussion

The effects on *S. avenae* exposed to the heavy metal Cd were examined by feeding on wheat plants grown in the Cd-treated soil. The concentrations in the leaves of wheat nonlinearly increased with increasing Cd levels in soil. Cd uptake by wheat might have been saturated because such high concentrations were present in the soil. Moreover, the data on Cd concentrations in the contaminated wheat revealed that the content was lower in plant leaves compared to that in the soil. This can be attributed to the conclusion put forward by Xiao (2006), who confirmed that the content of Cd in wheat initially increases, then decreases and increases again as the wheat grows. The heavy metal Cd mainly enriched in the roots of the wheat plant in the seedling stage (Xiao et al. 2006, 2009). Therefore, it is possible that the Cd levels of wheat seedlings used to feed the aphids in this experiment were lower in plant leaves than in the soil.

The results of this study indicated that development and reproduction of *S. avenae* were influenced by the heavy metal Cd, which supports previous research (Ruohomaki et al. 1996; Mousavi et al. 2003; Hayford and Ferrington 2005) that showed changes in developmental time, fecundity, and mortality of insects due to the heavy metal. In this study, the relative parameters of the life table, such as the net reproductive rate (*R_0_*), innate capacity of increase (*r*), and finite rate of increase (λ) were reduced. Fecundity and *m_x_* (the number of offspring produced by a female individual) were also negatively affected by Cd. Similar physiological changes were observed for *S. avenae* exposed to two types of heavy metals. Zhang and Zhao (2009) confirmed that *R_0_, r*, and λ of *S. avenae* decreased significantly with increasing concentrations of Zn (Zn and Cd).

It was worth noting that the most significant inhibition of survival and reproduction was observed at concentrations of 20 mg/kg and not the highest concentration (160 mg/kg). Our data revealed that *S. avenae* was more sensitive to Cd at concentrations of 20 mg/kg than other concentrations, which was different from previous reports by Kramarz and Stark (2003) and Xia et al. (2006), who concluded that the effect intensified with the increase of heavy metal concentration. It seems that cadmium is not the only factor of survival inhibition. There are other polluting inputs causing this inhibition.

Previous studies have shown the changes in activity of oxidative stress-related enzymes for superoxide dismutase and catalase, and detoxification enzymes for carboxyl-esterase (CarE) and glutathione transferase under treatment with heavy metals. For example, the activity of superoxide dismutase and catalase in *Boettcherisca peregrina* are restricted by Cd (Wang et al. 2006), and Zn and Cd increased the activity of carboxyl-esterase CarE and GST in *Poecilus cupreus* (Stone et al. 2002; Wilczek et al. 2003). In addition, the activity of CarE and CAT in *S. avenae* also changed under treatment with Zn (Zhang and Zhao 2009). Parallel studies are also performed on other insects (Zvereva et al. 2003). However, the changes in the activity of oxidative stress-related enzymes and detoxification enzymes in *S. avenae* exposed to Cd have not yet been examined. The activity of oxidative stress-related enzyme in wheat decreased with increasing Cd concentration (Zhang 2005; Wang 2008). Therefore, the research on the correlation between the enzyme activities in wheat leaves and in aphids is also necessary. The mechanistic changes in detoxification and oxidative stress-related enzyme activities in wheat leaves and aphids and their correlation would allow us to assess the impact of Cd on genetic and proteomic changes in *S. avenae* in the future.

## Abbreviations

Cd: cadmium
